# Genome expansion in bacteria: the curios case of *Chlamydia trachomatis*

**DOI:** 10.1186/s13104-015-1464-6

**Published:** 2015-09-30

**Authors:** Jon Bohlin

**Affiliations:** Department of Bacteriology and Immunology, Norwegian Institute of Public Health, Lovisenberggata 6, P.O. Box 4404, 0403 Oslo, Norway

## Abstract

**Background:**

Recent findings indicated that a correlation between genomic % AT and genome size within strains of microbial species was predominantly associated with the uptake of foreign DNA. One species however, *Chlamydia trachomatis*, defied any explanation. In the present study 79 fully sequenced *C. trachomatis* genomes, representing ocular- (nine strains), urogenital- (36 strains) and lymphogranuloma venereum strains (LGV, 22 strains), in three pathogroups, in addition to 12 laboratory isolates, were scrutinized with the intent of elucidating the positive correlation between genomic AT content and genome size.

**Results:**

The average size difference between the strains of each pathogroup was largely explained by the incorporation of genetic fragments. These fragments were slightly more AT rich than their corresponding host genomes, but not enough to justify the difference in AT content between the strains of the smaller genomes lacking the fragments. In addition, a genetic region predominantly found in the ocular strains, which had the largest genomes, was on average more GC rich than the host genomes of the urogenital strains (58.64 % AT vs. 58.69 % AT), which had the second largest genomes, implying that the foreign genetic regions cannot alone explain the association between genome size and AT content in *C. trachomatis*. 23,492 SNPs were identified for all 79 genomes, and although the SNPs were on average slightly GC rich (~47 % AT), a significant association was found between genome-wide SNP AT content, for each pathogroup, and genome size (*p* < *0.001*, *R*^2^ = *0.86*) in the *C. trachomatis* strains.

**Conclusions:**

The correlation between genome size and AT content, with respect to the *C. trachomatis* pathogroups, was explained by the incorporation of genetic fragments unique to the ocular and/or urogenital strains into the LGV- and urogential strains in addition to the genome-wide SNP AT content differences between the three pathogroups.

**Electronic supplementary material:**

The online version of this article (doi:10.1186/s13104-015-1464-6) contains supplementary material, which is available to authorized users.

## Background

Several studies have described the evolutionary process of genome reduction in which microbes undergo genome degradation as they become attached to a host, either in a parasitic or a mutualistic relationship [1]. The genomes of these microbes tend to be marked by dysfunctional DNA maintenance proteins and genetic drift which in turn lead to accumulated mutations in genes that are not important for the microbe’s existence [2]. Superfluous genes are eventually lost, since there appears to be an evolutionary drive in the direction of genome minimization [3]. Although exceptions exists [1], the genomes of these symbionts/mutualists are to a large extent AT-rich due to the mutational bias towards AT-richness [4]. A negative correlation has been found between genomic AT content and genome size in some prokaryotic phyla [5, 6] (i.e. genome size decrease with increasing AT content). However, it was recently shown that for strains within several microbial species there was a positive correlation between genomic AT content and genome size [7]. This correlation was linked to horizontal gene transfer (HGT), DNA uptake and/or recombination of AT rich sequences [7], but the species *Chlamydia trachomatis* seemed to be an exception since it could not be shown that foreign DNA had been integrated into the genomes of any of the sequenced strains [7].

*Chlamydia trachomatis* is a Gram-negative, obligate intracellular pathogen leading a parasitic lifestyle presumably only in humans [8]. Of the sequenced and assembled strains, the species’ AT content ranges from 58.5 to 58.7 % and the genome size from 1,038,310 to 1,083,890 bp. (See Additional file 1 for details). *C. trachomatis* has been divided into three distinct lineages, or pathogroups, based on serovars; one associated with trachoma (serovars A–C) another with urogenital infections (serovars D–K) and the third with lymphogranuloma venereum (LGV) and proctitis (serovars L1–L3) [8].

Although *C. trachomatis* is presumed to affect humans only, other animals may be infected by different species of *Chlamydia*. Since the focus of interest here is genome expansion in *C. trachomatis* the reader is referred elsewhere for more information concerning other species of *Chlamydia* [8, 9].

Hence, the aims of the study were to scrutinize the positive association between AT content and genome size in *C. trachomatis* to shed more light on the evolutionary processes involved.

## Results

### Exploration of the different pathogroups

**Fig. 1:**
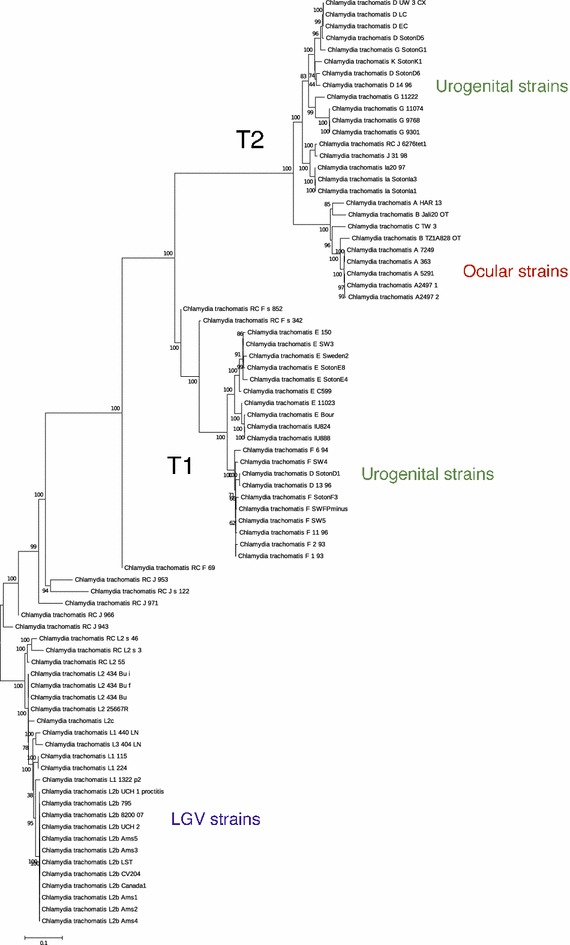
SNP-based phylogenetic tree. The *figure* shows a phylogenetic tree, rooted by the lymphogranuloma venereum (LGV)-strains, which presumably are most similar to the last common ancestor of the 79 *C. trachomatis* genomes depicted. The strains are divided into two biovars: the LGV- and urogenital strains. The urogenital strains are further split into two lineages* T1* and* T2*. The* T2* lineage is again split into the urogenital- and ocular strains

**Fig. 2:**
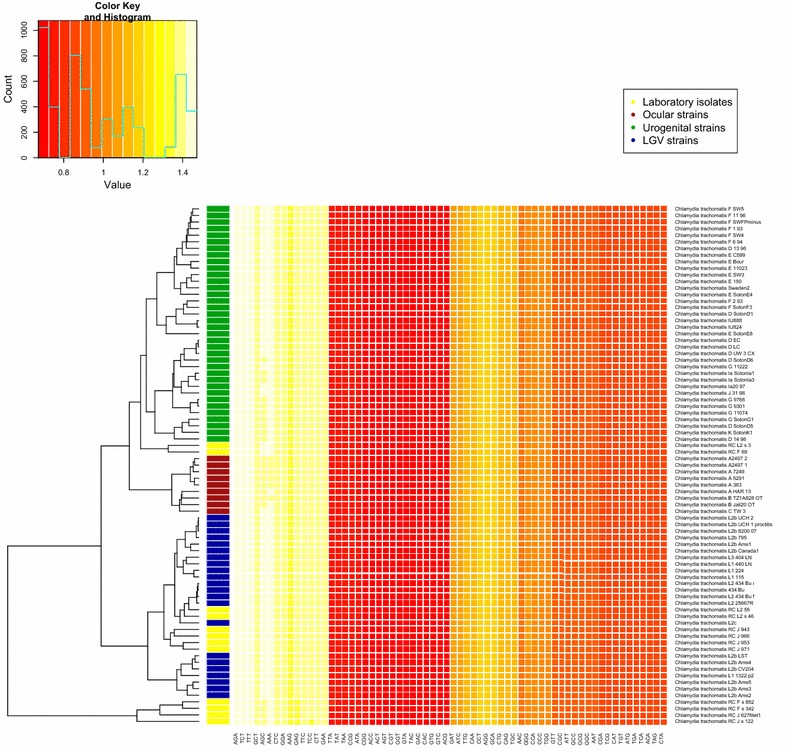
Genome-based taxonomic assessment of *C. trachomatis* strains. The heatmap shows a two-way hierarchical cluster analysis performed on the estimated zero’th order Markov model based trinucleotide frequencies in 79 *C. trachomatis* genomes. The leftmost colored column indicates pathogroup: *red* trachoma (A–C) serovars, *green* urogenital (D–K) serovars, *blue* LGV/proctitis (L1–L3) serovars and *yellow* recombinant (in vitro) isolates. Except for the recombinant isolates, which have genomes consisting of a mixture of genomes from multiple *C. trachomatis* strains from different serovars in addition to foreign genetic elements from other species, the pathogroups cluster perfectly together. A slight, but marked, division can also be observed within the urogenital *C. trachomatis* serovars (*green*)

Since *C. trachomatis* taxonomy is complicated by the discovery that substantial parts of the outer membrane gene (*ompA*), much used for taxonomic assessment of *C. trachomatis* strains into different serovars, has often undergone HGT with other *C. trachomatis* strains [10], additional tests to assess *C. trachomatis* phylogeny were carried out. This included genome-wide single nucleotide polymorphism (SNP) based phylogenetic analysis in addition to a hierarchical cluster analysis based on whole-genome trinucleotide frequencies normalized by the respective nucleotide frequencies (trinucleotide zero’th order Markov model) on 79 *C. trachomatis* genomes [11]. A lot can also be said about *C. trachomatis* phylogeny by scrutinizing the different strains corresponding plasmids, but since the focus of the present work is genome expansion with respect to the main chromosome the interested reader is referred to the article by Harris et al. which gives a thorough account on the subject [12]. From both Figs. 1, 2, respectively describing the SNP based- and hierarchical cluster analysis, it can be seen that the pathogroups cluster perfectly together (the yellow laboratory isolates are *C. trachomatis* isolates from the three pathogroups artificially manipulated in the laboratory) indicating that the serovars associated with each pathogroup are subjected to similar selective pressures [2, 13]. Four of the included recombinant laboratory isolates contained a genome island (GI) with antibiotic resistance genes also identified in the pig pathogen *C. suis* [14, 15]. Both Figs. 1, 2 show that there is a slight division within the urogenital strains (colored green), which is also supported by previous work [12, 16]. While pattern differences from trinucleotide frequencies can be observed in Fig. 2, the SNP-based phylogenetic tree in Fig. 1 suggests a greater division amongst primarily the E and F serovars (including *C. trachomatis* D SotonD1 and D 13 96 strains) of the urogenital strains and the D, G, I, J and K serovars, which are closer to the trachoma strains of the A–C serovars than the E and F serovars.

### Genome size, base composition and mutational bias

**Fig. 3:**
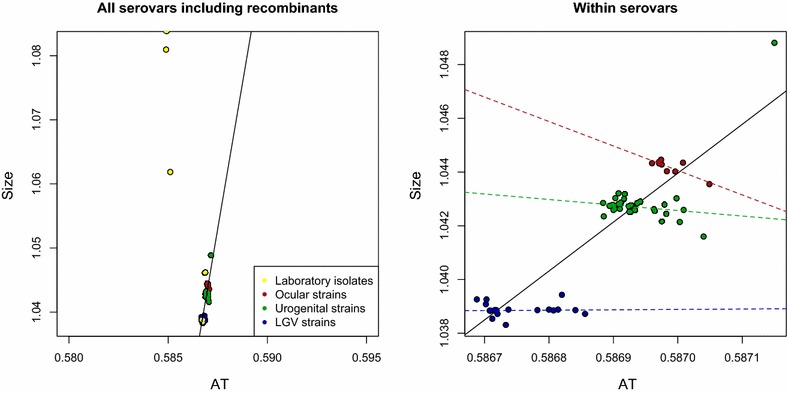
Size versus AT content in all *C. trachomatis* strains. The *left panel* shows the genomic fraction of AT content (*horizontal axis*) in 79 *C. trachomatis* genomes, colored according to pathogroup, regressed against genome size in mb (*vertical axis*), together with the estimated regression coefficient (*black line*). The right panel shows a close up of the *left panel* with the 12 recombinant in vitro strains removed. The *dotted line*, colored according to pathogroup, represent the estimated regression coefficients for each pathogroup

Figure 3 demonstrates a positive correlation between genome size and AT content in the *C. trachomatis* strains (average genome size and AT content for all pathogroups and laboratory isolates can be found in Table 1), and that this association is linked to the different pathogroups (*p* < *0.001*). Within the pathogroups, however, no such association was found (*p* ~ 0.54, *p* ~ 0.74 for the urogenital- and LGV pathogroups containing 36 and 22 strains, respectively) with the exception of the strains in the pathogroup linked with trachoma (*p* < 0.001). The trachoma pathogroup however consisted of nine strains only and the result must therefore be considered as preliminary at the moment and will not be further discussed here.

**Table 1:** Average genome size and AT content in the different pathogroups

Pathogroup	Number of strains	Average genome size (mb) (±95 % CI)	Average AT content (±95 % CI)
Ocular strains	9	1.0441 (1.0439–1.0444)	0.586987 (0.586966–0.587008)
Urogenital strains	36	1.0428 (1.0425–1.0432)	0.586941 (0.586923–0.586959)
LGV strains	22	1.0388 (1.0387–1.0389)	0.586746 (0.586723–0.586769)
Laboratory isolates	12	1.0529 (1.0407–1.0651)	0.586163 (0.585589–0.586718)
All 79 genomes	79	1.0434 (1.0415–1.0453)	0.586772 (0.586675–0.586870)

**Fig. 4:**
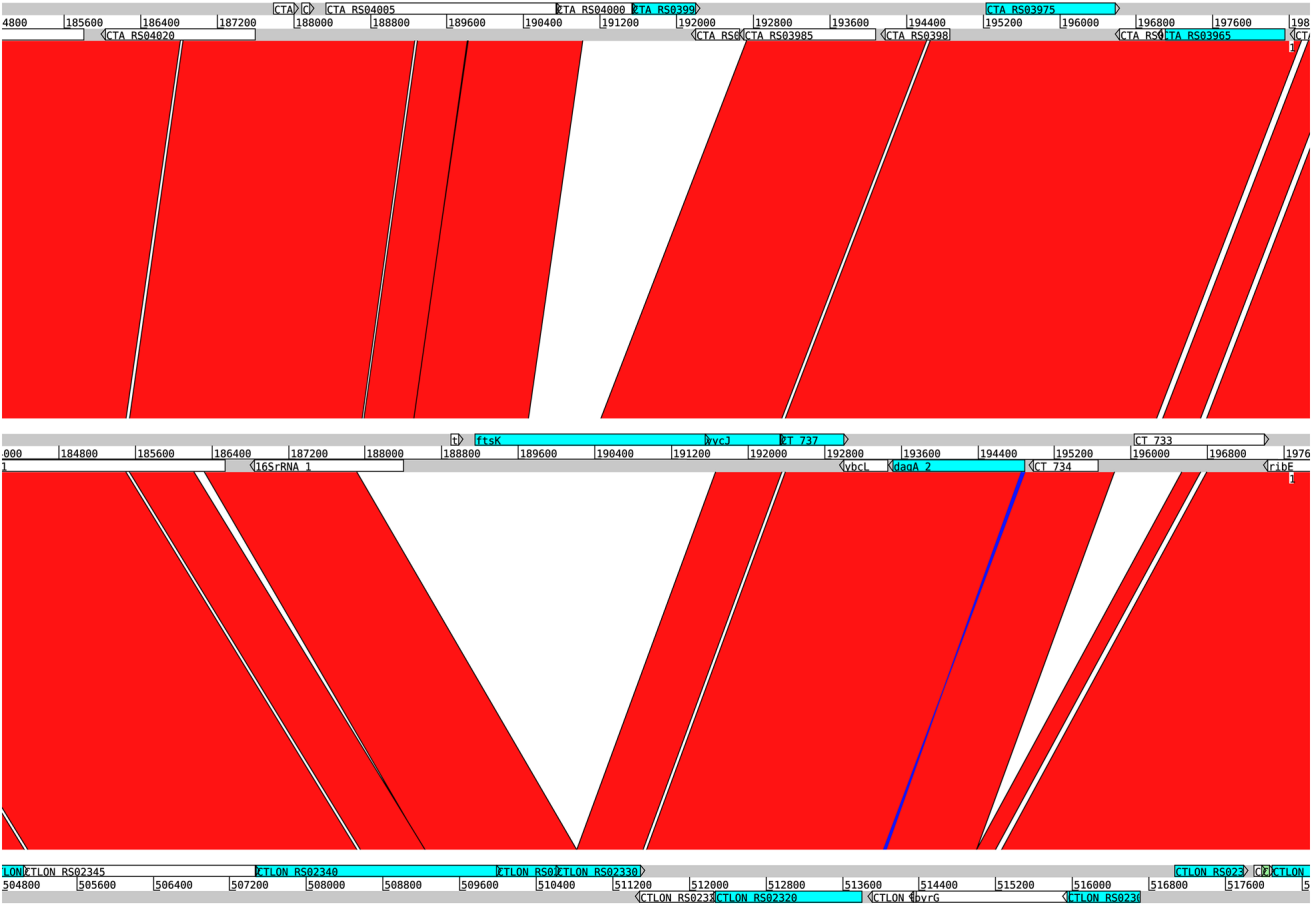
ACT comparison of one strain from each pathogroup. The *figure* shows an ACT comparison between *C. trachomatis* A/HAR 13 (*top*), *C. trachomatis* D/UW-3/CX (*middle*) and *C. trachomatis* L2b UCH1 (*bottom*). The *figures* also show designated genes for all strains as well as genomic positions. The *red* regions indicate presence of genetic regions in all genomes, while the white regions designate absence of genetic regions in one or more of the genomes

**Fig. 5:**
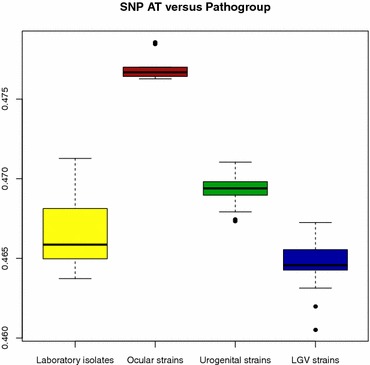
SNP AT content versus pathogroup. The *box plot* shows the AT content from 23,492 SNPs (*vertical axis*) in the LGV- (22), urogenital (36) and ocular strains (9), as well as for the laboratory isolates (12)

**Fig. 6:**
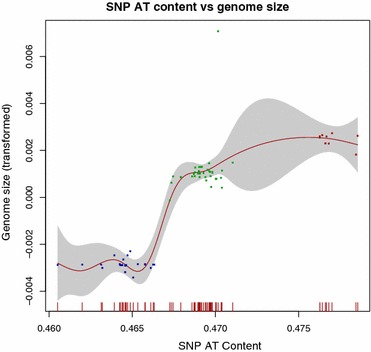
SNP AT content versus genome size. The *graph* shows a generalized additive model (GAM) with genome size in 67 *C. trachomatis* genomes as the transformed response (*vertical axis*) and SNP AT content, estimated using a spline, as the explanatory variable (*horizontal axis*). The genomes are colored according to pathogroup: *red* ocular strains, *green* urogenital strains and *blue* LGV strains

The genetic regions unique to each pathogroup have been described previously [17], and a detailed visualization can be observed from the ACT-comparison [18] in Fig. 4 between the trachoma strain *C. trachomatis* A/HAR 13 (NC 007429.1) the urogenital strain *C. trachomatis* D/UW-3/CX (NC 000117.1) and the LGV strain *C. trachomatis* L2b UCH1 (NC 010280.2). The genes particular to each pathogroup are shown in Table 2. Table 3 describes the average AT content and the size of these genetic regions unique to each pathogroup. It can be seen from Table 3 that the genomic fragments unique to both serovars A–C and D–K, and not found within the LGV clade, are more AT rich than the LGV strains on average (see Table 1). Moreover, the genetic fragments predominantly found within serovars A–C are, in fact, slightly more GC rich than the urogenital strains on average, as well as the strains in serovar A–C, implying that the incorporation of these genetic fragment into the urogenital strain would not explain the AT content difference between the urogenital and ocular pathogroups (see Table 4). Furthermore, Table 4 indicates that the average AT content and the size of the genomic fragments from the different serovars can, in general, not fully explain the AT content differences between the three pathogroups. To elucidate this conundrum further mutational biases were analyzed from the genome-wide SNP analyses of which the phylogenetic tree in Fig. 1 is based on. The SNP-based phylogenetic tree demonstrates that there are differences with regards to mutational biases, and that these correspond well with the established pathogroups [12]. Moreover, Fig. 5 demonstrates that SNP AT content differs significantly between the pathogroups (*p* < 0.001), with the possible exception of the laboratory isolates, although varying substantially in size have SNPs and AT content more similar to the strains from the pathogroups they were originally chosen from (*p* ~ 0.05 to *p* ~ 0.002 with the LGV and urogenital pathogroups, respectively) [14]. Nevertheless, as can be seen from Fig. 5, the average SNP AT content is highest for the trachoma strains, second highest for the urogenital strains and lowest for the LGV strains, which also correspond with the average genome size of each pathogroup (of course, with the exception of the artificially manipulated laboratory isolates). Figure 6 demonstrates that a strong association between genome size and SNP AT content was observed (*p* < 0.001, *R*^2^ = 0.86), although this association is not linear. The incorporation of the average SNP AT content differences between the pathogroups to the average AT content of the genomes in serovars D–K and L1–L3, together with the average AT content of the corresponding genetic fragments found in serovars D–K and A–C, resulted in slightly higher estimates of genomic AT content in serovars L1–L3 and D–K than observed in serovars D–K/A–C. As can be seen from Table 4 the differences are small for each of the pathogroups and this could well be explained by lack of amelioration [19] and/or the widespread recombination reported in the urogenital- and ocular clades [12, 16, 20, 21]. Indeed, recombination within the urogenital biovar (including both T1 and T2 clades, as shown in Fig. 1) may have occurred for as much as 30–40 % of the *C. trachomatis* genome [16, 22]. Thus, the discrepancies between AT content and genome size not accounted for by the genomic fragments unique to the trachoma- and urogenital strains, in addition to differences in SNP AT content, are attributed to recombination events.

**Table 2:** Genes and proteins unique to the urogenital biovar

Gene fragment in serovars	DNA sequence	Protein sequence
Serovar A–C	Part of cytotoxin gene (AY647992.1)	Putative cytoadherence factor (CAX09718.1)
		DUF3491 (pfam11996)
Serovar A–C/D–K	Cytotoxic genes (homologous to AY647998.1)	TcdA/TcdB catalytic glycosyltransferase domain (pfam12919)
		TcdB toxin N-terminal helical domain (pfam12918)
		DUF3491 superfamily (pfam11996)

**Table 3:** Average size and AT content of fragments unique to pathogroups

Pathogroup	Isolates#	Average size (bp) (95 % CI)	Average AT content (95 % CI)
Genetic region not found in LGV strains	9	5789.7 (5753–5824.3)	0.601663 (0.600759–0.602566)
Genetic region exclusive to the ocular strains	9	1676.1 (1675.9–1676.4)	0.586357 (0.586357–0.586730)
Genetic region found in both ocular and urogenital strains	36	4445.9 (4443.3–4448.6)	0.609864 (0.609641–0.610087)

**Table 4:** AT content estimation of serovars A–C/D–K

Genomic fragment + serovar	Estimated AT content from fragments	Estimated AT content + SNP AT	Observed average AT content
Sr L1-L3 + fragment Sr A–C	0.5868286 (0.5868004–0.586857)	0.5871145 (0.5870662–0.587163)	0.586987 (0.586966–0.587008)
Sr L1-L3 + fragment Sr D–K	0.5868445 (0.5868206–0.5868684)	0.5869537 (0.5869141–0.5869932)	0.586941 (0.586923–0.586959)
Sr D-K + fragment Sr A–C	0.586940 (0.5869221–0.5869586)	0.5871161 (0.587082–0.5871508)	0.586987 (0.586966–0.587008)

Estimated average AT content (95 % CI) for Serovars L1–L3 and D–K after the inclusion of genetic fragment average AT content (95 % CI) unique to Serovars A–C and/or D–K (column 2), as described in column 1. In column 3, average SNP AT content differences (95 % CI) between the pathogroups are included to the estimates in column 2. Column 4 describes average AT content (95 % CI) for all nine strains in Serovars A–C (top and bottom row) and the 22 strains in Serovars D–K (middle row)

## Discussion

### Evolutionary analyses of the *C. trachomatis* strains

Compelling evidence has been put forth that there has been an early split in *C. trachomatis* from the LGV strains and the urogenital strains into two distinct clades, or biovars [12]. Figure 1 indicates that the passing of this coalescent event must be one of the earliest known occurrences of a split within *C*. *trachomatis*. Within the LGV clade there has been recorded only sporadic re-combination events and even fewer such events between the LGV and urogenital strains [16, 22]. Hence, the LGV clade has, at least until now, been largely isolated from the urogenital strains [16, 23]. Within the urogenital strains however, several splitting events have taken place, in addition to numerous recombination events [12, 16, 22]. The earliest splitting within the urogenital clade seems to have resulted in two new minor clades (T1 and T2) both comprising predominantly urogenital strains [12, 22, 23]. Figure 3 shows a distinct size difference between the *C. trachomatis* A–C serovars and the urogenital pathogroup, suggesting an even more recent split within the urogenital T2 clade into the ocular pathogroup. That the strains from the A–C serovars have had a recent common ancestor with the T2 clade can be seen from the SNP based phylogenetic tree in Fig. 1 as well as in numerous studies [12, 16, 23]. That the ocular strains have emerged only once from the T2 clade, as suggested by Harris et al. [12] seems therefore to be supported by the approximately 1670 bp genetic fragment predominantly found within the A–C serovars. Interestingly, this genetic fragment was found to be GC-rich and increased GC content in genes has recently been associated with increased fitness [24, 25].

The LGV strains do not appear to have undergone any further major splits, and the strains’ genomes seem to be more homogeneous genome-wise than the strains within the urogenital clades [12].

### The last common ancestor of the LGV- and urogenital strains

Due to the genome-wise homogeneity within the LGV clade it is tempting to speculate that these strains are more similar to the last common ancestor of both the LGV and urogenital strains. Within the urogenital clade however, there is wide-spread recombination [12, 16, 22, 23] indicating far more genomic plasticity than what has been shown for the LGV clade [16, 22]. Horizontal transfer of DNA from distantly related bacteria to *C. trachomatis* does not seem to have taken place, at least not to a large extent. Indeed, the pan- and core genomes of the *C. trachomatis* strains have been found to be of similar size suggesting that genetic exchange takes place mostly within the same species [16]. In fact, as was noted above, genetic exchange appears to predominantly occur within the same biovar [12], which suggests that the strains within the urogenital clade have changed considerably more genome-wise with respect to the last common ancestor of both urogenital- and LGV clades.

### Analyses of *C. trachomatis* SNP AT content

From Fig. 5 it can be observed that the SNPs from the ocular strains are more AT rich than those from the urogenital strains, which in turn are more AT rich than those from the LGV strains. The largest difference detected in SNP AT content between the three lineages is within the ocular strains. This could mean that the split from the T2 clade mentioned above may be a consequence of a shift in environmental conditions leading to a substantial increase in genetic drift and/or a considerable reduction in population size [2]. In this regard it is interesting to recall the fact that the 23,942 SNPs identified genome-wide for all 79 *C. trachomatis* genomes were slightly GC rich (see Fig. 5). The GC-richness of the SNPs may indicate that *C. trachomatis* is, in general, subjected to selective constraints [24–26]. Since increased GC content has been associated with intensified selective pressures [24, 25] the larger genome size of the progressively more AT rich genomes of the urogenital- and ocular strains, respectively, could indicate that the increase in genome size in these strains are in fact a consequence of reduced selective constraints that allowed the strains to incorporate the genetic fragments [27]. Or, alternatively, the split into urogenital- and ocular pathogroups is so recent that the consequences of relaxed selective pressure has not started to result in reduced genome sizes. Nevertheless, the substantial increase in SNP AT richness for the ocular strains may support a recent dramatic split from the T2 clade [12].

### *C. trachomatis* genome size and genomic fragments unique to the urogenital clade

Genome size in *C. trachomatis* was found to correlate with genomic AT content. A more detailed investigation, as can be seen in Fig. 3, revealed that the correlation between genome size and AT content was linked to the different pathogroups (excluding the artificially manipulated laboratory isolates). The correlation between AT content and genome size in the *C. trachomatis* pathogroups was largely explained by the genome size and the AT content of the genetic fragments unique to serovars A–C and D–K (see Table 4). On average, however, the addition of AT content from these genetic fragments to the genomic AT content of the LGV- and urogenital pathogroups resulted in slightly less AT rich genomes than what was observed from the strains incorporating these fragments. The average AT content differences between the pathogroups from the 23492 SNPs were therefore also incorporated into the AT content estimates of the pathogroups lacking the genetic fragments unique to servars A–C/D–K. This resulted in slightly more AT rich pathogroups on the whole suggesting that both mutational biases, as described by the SNPs, as well as the incorporation of the genetic fragments unique to the urogenital- and ocular pathogroups explained the correlation between genome size and AT content in *C. trachomatis*. The lack of correlation between AT content and genome size within the LGV- and urogenital lineages appears therefore to be explained by, first and foremost, the absence of the genetic fragments. The difference in AT content within all three lineages, as can be observed from both Figs. 3, 5, seems to be described by smaller recombination events and mutational biases [21, 22]. The genetic fragments, or lack thereof, as in the case of the LGV strains, have previously been linked to symptoms particular to each pathogroup, i.e. urogenital infections (serovar D–K) and trachoma (serovars A–C) [20, 23, 28].

### The origins of the genetic fragments unique within the *C. trachomatis* urogenital biovar

The genetic fragments particular to the trachoma and urogenital strains discussed here are widely present in other *Chlamydia*-species known to infect other animals [8, 9, 14, 15]. Since the genetic fragments unique to the urogenital and/or oculars strains, described in the present work, are present in several other *Chlamydia* species a natural question is why they are not present in the LGV strains (and some only partly in the urogenital strains). One possibility is that the human infecting urogenital and LGV strains have undergone genome reduction from a common ancestor with the ocular strains. Since all the genetic fragments present in the ocular and urogenital strains are extant in several other *Chlamydia* species it is conceivable that they have been lost and re-gained in the *C. trachomatis* strains. However, this is difficult to document and if so this cannot have occurred in recent times since a BLAST search [29] with the genetic fragments unique to *C. trachomatis* ocular- and urogenital strains were found to have a base composition substantially different to that found in the other *Chlamydia* species [8, 9]. It is also at odds with recent genomic research on *C. trachomatis*, which provide compelling evidence that the urogenital and LGV biovars shared a last common ancestor [12, 16]. Hence, a more plausible scenario is that the genetic fragments exclusive to serovars A-C and the urogenital strains have been attained due to more recent recombination. This could indicate that the genetic fragments found only in the urogenital- and ocular pathogroups are present in other yet to be discovered *C. trachomatis* strains. Indeed, in Figs. 3, 5 one outlier from the urogenital lineage can be observed. This outlier was identified as *C. trachomatis* E SotoenE8. The strain appears to have a duplicated region in the plasticity zone [17] (approximate genomic position: 190,300–195,300, found to contain the proteins: PhnP, GloB, AlsT with the additional protein domains identified: SET, ABC_ATPase, PEBP_bact_arch, Ftsk_gamma) also present in the other urogenital strains. Interestingly, the same genetic fraction consisting of approximately 1670 nucleotides predominantly found in the ocular strains was also present in this urogenital strain. Yet, based on whole genome SNPs, the *C. trachomatis* E SotoenE8 strain cluster with the urogenital T1 lineage (see Fig. 1). This may indicate that the genetic fragment predominantly found in the A–C serovars is also available for the D–K serovars suggesting that the acquisition of this genetic fragment may not alone sufficiently explain the splitting of the T2 clade into the ocular pathogroup.

### DNA uptake in *Chlamydia* sp.

An example of DNA uptake in *Chlamydia* sp. was observed in the previously described pig pathogen *C. suis* which has been found to carry a GI with antibiotic resistance genes [8]. It is believed that *C. suis* may have picked up the GI as a consequence of co-infection with the γ-proteobacterium *Aeromonas salmonicida* and the ε-proteobacterium *Helicobacter pylori* [15]. Although many co-infecting bacteria may not be directly pathogenic to their hosts the case of *C. suis* shows that they can provide genes conferring resistance to tetracyclines, even though they belong to different phyla and have very different base compositions (The average AT content of *A. salmonicida* is ~41.5 %, compared to ~58 % for *C. suis*) [14]. The *C. trachomatis* laboratory isolates, mentioned previously, shows that gene transfer can take place between *C. trachomatis* and distantly related bacteria as well and therefore that the human pathogen can potentially be an even more serious threat to public health; not only in developing countries but also in developed countries as well since *C. trachomatis* infections are, in general, treated with tetracycline-based antibiotics [15, 28].

## Conclusion

The correlation between genome size and AT content in *C. trachomatis* cannot be explained by the incorporation of AT-rich genetic fragments alone. Additionally accounting for SNP AT content differences, however, largely resolves the differences in AT content observed between the pathogroups. Whether the association between SNP AT content and genome size is due to relaxed selective forces remains to be identified.

## Methods

All genomes were downloaded from NCBI/GenBank: http://www.ncbi.nlm.nih.gov/genome [30]. Additional file 1 contains all NCBI accession numbers. The genomes of the strains: *C. trachomatis* L2b Canada2 (NC020932), *C. trachomatis* E 12 94 (NC022108) and *C. trachomatis* J 27 97 (NC022110) were removed because of sequence errors. Genomic AT content and genome size were calculated using in-house scripts. The heatmap in Fig. 2 is based on whole-genomic trinucleotide frequencies for each genome normalized by the respective genome’s nucleotide frequencies:

$$ \frac{{f_{i} (XYZ)}}{{f_{i} (X)f_{i} (Y)f_{i} (Z)}} .$$

*f*_*i*_ designates the genomic frequencies of overlapping trinucleotides *XYZ* or nucleotides *X*, *Y* or *Z* for genome *i*. Hierarchical cluster analysis, based on average linkage and Euclidean distance, was carried out on all possible combinations of trinucleotides (64 in total) for each of 79 *C. trachomatis* genomes [11]. MAUVE [31] was used to identify SNPs for all C. *trachomatis* genomes and the foreign genetic regions were identified using MAUVE and the Artemis Comparison Tool (ACT) [18]. BLAST [29, 30] was used on the NCBI “nr”-database [30] to extract the genomic fragments from all *C. trachomatis* strains as well as to search other *Chlamydia* sp. for these fragments. BLAST was also used to prepare the genomes of *C. trachomatis* A/HAR 13 (NC 007429.1), *C. trachomatis* D/UW-3/CX (NC 000117.1) and *C. trachomatis* L2b UCH1 (NC 010280.2) for ACT. The SNP-based phylogenetic tree was based on maximum likelihood estimation using the MEGA 6 software package [32], while the optimal nucleotide substitution matrix was determined using the “ape” package in R [33]. The nucleotide substitution matrix obtaining the lowest AIC score [34] was GTR I + G, which was subsequently used in MEGA 6 for the construction of the maximum likelihood-based phylogenetic tree. The SNP sequences were aligned with MAFFT using automatic settings (selecting “rough alignment”) [35].

Due to few samples, outliers and indications of heteroscedasticity, correlation/association between genomic AT content and genome size was performed using “MM” type regression [36], from the “robust” package in R, for increased reliability. For the same reasons, statistical comparisons between pathogroups and genome size was performed using a modified version of Tukey’s test with the method described by Hothorn et al. [37], and implemented in the R package “multcomp”, incorporating the vcovHC3 sandwich estimator [38] to compensate for the heteroscedastic variance. The average values found in Tables 1, 2 and 4 were computed using Welch’s *t* test with 95 % confidence intervals (95 % CI). The non-linear association observed between SNP AT content in the 79 *C. trachomatis* genomes and genome size was modeled using a spline-based generalized additive model (GAM) [39] with genome size as the response variable and AT content as the explanatory variable. Details regarding the statistical estimations can be found in Additional file 2. The estimation of AT content change in the LGV and urogenital pathogroups was calculated by adding the average AT content of the genetic fragment to the average AT content of the genomes in the pathogroups of which the genetic fragments were missing and divide by the total number of nucleotides (i.e. average genome size of the genetic fragments in question and *C. trachomatis* genomes, respectively). The smallest and largest genome sizes as well as the highest and lowest AT content were calculated to produce the minimum/maximum intervals for both genome fragment and genome-wide AT content and genome size. An analogous procedure was carried out to account for AT content differences due to SNPs between the pathogroups; the 23492 SNPs were divided by the corresponding average pathogroup genome size and multiplied by the difference in SNP AT content between the pathogroups. The resulting estimates were added to the above-mentioned calculations regarding the genetic fractions unique to the A–C/D–K serovars. All statistical assessments were carried out in R [40].

## Additional files

10.1186/s13104-015-1464-6 Information regarding the *C. trachomatis* strains used in the study.
10.1186/s13104-015-1464-6 Results from statistical models.
